# Long-Term Health Risks for Children and Young Adults after Infective Gastroenteritis

**DOI:** 10.3201/eid1609.081665

**Published:** 2010-09

**Authors:** Rachael E. Moorin, Jane S. Heyworth, Geoffrey M. Forbes, Thomas V. Riley

**Affiliations:** Author affiliations: The University of Western Australia, Crawley, Western Australia, Australia (R.E. Moorin, J.S. Heyworth, T.V. Riley);; Royal Perth Hospital, Perth, Western Australia, Australia (G.M. Forbes)

**Keywords:** Gastrointestinal infections, sequelae, data linkage, long-term health risk, children, adults, bacteria, research

## Abstract

To quantify the risk and types of sequelae attributable to prior enteric infections, we undertook a population-based retrospective cohort study using linked administrative records. The risk for first-time hospitalization for sequelae was modeled by using Cox proportional regression analysis controlling for other health and sociodemographic factors. We identified a significant increase of 64% in the rate of first-time hospitalization for sequelae for persons with prior enteric infections: 52% for intragastrointestinal sequelae and 63% for extragastrointestinal sequelae compared with first-time hospitalization for those without prior infection. Extragastrointestinal sequelae occurred predominantly during the first 5 years after first-time enteric infection. In contrast, most intragastrointestinal sequelae occurred >10 years later. Infective gastroenteritis during childhood or adolescence increases the risk for first-time hospitalization for intragastrointestinal and extragastrointestinal disease over the 2 decades after first-time enteric infection, highlighting the importance of identifying ways of reducing the incidence of such infections.

## Abstract 2

TOC Summary: Prior episodes increase risk for long-term adverse health effects.

Gastroenteritis is a common illness worldwide and has a considerable effect on the public health of communities and health systems that provide care. In developing countries, gastrointestinal infection is a major cause of death, claiming ≈2 million lives each year among children <5 years of age (*1*). By contrast, most episodes of gastroenteritis in industrialized nations do not cause serious, immediate, adverse sequelae but remain common, especially in the young (*2*,*3*). In addition to the immediate health concerns associated with gastroenteritis, subsequent medium- to long-term adverse sequelae have been described. A range of gastrointestinal, rheumatologic, neurologic, and skin and lung conditions have been associated with previous exposure to enteric infections (*4*–*13*).

Most of these data are from case reports and small-sample cross-sectional studies; however, several recent short-term longitudinal studies have provided estimates of the incidence of adverse health events after enteric infections (*8*,*14*,*15*). These studies suggest that the increase in risk for sequelae is considerable. For example, in a follow-up cohort study of a community exposed to a waterborne disease outbreak, the relative risks for chronic gastrointestinal symptoms, arthralgia, and psychiatric conditions were 2.4, 1.4, and 2.0, respectively (*16*). However, the long-term population-based extent of sequelae from prior enteric infection has remained unclear because previous studies have not adjusted for confounding variables, follow-up was short-term (*8*), evaluations were taken when populations were exposed to an outbreak of water-borne disease (*8*,*14*), and adverse events were either identified by self-reporting (*8*,*14*) or compared with expected, but not measured, rates of events in the general population (*15*).

The Western Australia Data Linkage System (WADLS) provided a unique opportunity to undertake a robust, long-term, longitudinal study of the sequelae associated with notifiable enteric infections in the general population. This system enables capture of health events in persons previously exposed and not exposed to an enteric infection. Our goal was to quantify the rate, risk, and type of sequelae attributable to previous childhood and adolescent exposure to enteric infections that lead to hospitalization, controlling for other health and sociodemographic factors.

## Methods

This retrospective, population-based, longitudinal cohort study linked routinely collected administrative records from the Western Australian notifiable infectious diseases database (NIDD) with data contained in the Western Australian hospital morbidity data system (HMDS). Western Australian death notifications routinely collected under the Western Australian State government statute were also linked.

### Study Participants and Sources of Data

The cohort comprised all persons having a Western Australia birth notification during January 1, 1985–December 31, 2000. For each person, the following records were extracted by the WADLS: 1) NIDD records of any enteric infection from birth through December 31, 2007, and including encrypted patient identification, sex, age at notification, date of notification or onset, species causing enteric infection, and residence recorded as postal code at notification; 2) HMDS records of all separations (discharge from hospital) from any Western Australia hospital (public and private) from December 31, 2007, and comprising encrypted patient identification, age at hospitalization, sex, Aboriginality, date of admission, and International Classification of Diseases, 9th Revision, code for principal and additional diagnoses; and 3) any death notification for the person, comprising encrypted patient identification, sex, age at death, and date of death.

### Definition of Prior Infection

Each person was assigned to 1 of 2 mutually exclusive groups. The exposed group was defined as persons with any past enteric infection. More specifically, these were past infections notified on the NIDD or a past hospitalization for an enteric infection recorded on the HMDS where no NIDD notification was present. The unexposed group (those with no prior infection) comprised persons for whom the NIDD and HMDS had no notifications pertaining to any form of gastroenteritis.

For the exposed group, the date of past infection was the date of onset recorded on the NIDD notification or the date of admission recorded on the HMDS for a first-time enteric infection. Using a birth cohort methodology, the unexposed group (no prior infection) would be expected to be followed up for longer (birth to outcome) than the exposed group (prior infection to outcome). To reduce this bias, each person in the unexposed group was given a proxy date of prior infection achieved by randomly assigning the exposure dates observed in the exposed group to nonexposed persons.

### Definition of Outcome

We were interested in outcomes (sequelae) that resulted in hospitalization. Therefore, the outcome was defined as a first-time hospitalization and a diagnosis of any sequelae of interest recorded as either the principal or co-diagnosis at any time from birth to death or end of follow-up. The sequelae of interest were further divided into 2 broad groups (intraintestinal and extraintestinal conditions) (Table A1).

To differentiate persons with a first-time hospitalization for the sequelae of interest after a NIDD notification (incident cases) from those with a previous hospitalization for a sequelae before exposure (prevalent cases), we examined HMDS records dating back to birth. Prevalent cases were excluded from the study. For each person, the follow-up time, or time at risk for sequelae (years, or part thereof) was enumerated from the date of prior infection, or proxy date of prior infection for unexposed persons, to admission date for the sequelae of interest, date of death, or December 31, 2007 (end of follow-up), as appropriate.

### Determination of Socioeconomic Status and Residence

Socioeconomic status and residence were also determined from existing records. Published Socio-Economic Indexes for Areas (Index of Relative Social Disadvantage) (*17*) and Accessibility/Remoteness Index of Australia scores (*18*) were mapped to the postal codes of persons at birth.

### Presence of Preexisting Concurrent Conditions

The presence of preexisting concurrent conditions was identified by using the International Classification of Diseases, 9th Revision, codes for hospital separations. The Multipurpose Australian Comorbidity Scoring System (*19*) was used to determine any hospitalization for a concurrent illness for each person at the outcome, death, or end of follow-up.

### Analysis

Data were analyzed by using SPSS software, version 14 (SPSS Inc., Chicago, IL, USA). Incidence rates for individual enteric infections and types of sequelae were calculated by using the number of events as the numerator and the follow-up time as the denominator.

After determining that the structure of the data was appropriate by testing the proportional hazards assumption (*20*), we used the Cox proportional hazards regression model to conduct survival analyses. We examined the risk for first-time hospitalization for any sequelae, intragastrointestinal sequelae, and extragastrointestinal sequelae over time by using Cox proportional hazards regression models, in which we compared the risk for first-time hospitalization among persons with prior enteric infection with that of those who had no prior infection. These models were adjusted for factors that may have influenced the probability of first-time hospitalization occurring, i.e., sex, indigenous status, year of birth, age at exposure or proxy, singleton versus multiple birth status, weight at birth, hospital birth versus nonhospital birth, mother’s region of birth, father’s region of birth, socioeconomic status, accessibility to services, and previous hospitalization for comorbidity.

When possible, Cox proportional hazards models were also constructed separately by type of enteric infection. To determine which sociodemographic or disease factors were influential in sequelae development, separate survival models were also constructed for exposed and unexposed groups.

Attributable risk percent was used to estimate the proportion of sequelae for which prior exposure to an enteric infection was a component cause. This attributable risk was calculated as the adjusted rate ratio (obtained from the Cox proportional hazards regression model) minus 1, divided by the adjusted rate ratio, multiplied by 100. Thus, we estimated the percentage of first-time hospitalizations for sequelae in the exposed cohort that were attributable to being previously exposed to an enteric infection after controlling for known potential confounders.

### Ethical Approval

The study was approved by The University of Western Australia’s Human Research Ethics Committee. All data were deidentified before being provided to the researchers.

## Results

### Study Participants

Of the 336,401 persons who met the inclusion criteria, 23,477 (7%) had at least 1 notification for an enteric infection during the 22-year study period. Similar proportions (<3% difference) of male patients, nonindigenous persons, singleton births, and hospital births were found in both groups (Table 1). For those with prior enteric infection, small birth-weight babies <3,000 grams were overrepresented (p<0.0001); normal birth-weight babies 3,001–4,000 grams were underrepresented; and disadvantaged persons (socioeconomic status and accessibility to services; p<0.0001), persons having prior comorbidity (p<0.0001), and persons having prior hospitalization (p<0.0001) were overrepresented. The median year of birth for those with prior enteric infection was 1994, compared with 1992 for those with no prior enteric infection; however, the mean age at exposure or proxy was similar for both groups (2.9 years and 2.4 years, respectively). Mean survival to a first-time hospitalization for any sequelae, death, or end of follow-up for persons with and without prior enteric infection was 8.6 and 11 years, respectively (p<0.0001).

**Table 1:** Sociodemographic characteristics and measures of preexisting health status for those with and without history of enteric infection, Western Australia, Australia, January 1, 1985–December 31, 2000

Characteristic	History, no. (%), n = 23,477	No history, no. (%), n = 312,924	% Difference*
Male sex	12,297 (52.4)	156,873 (50.1)	2.3
Not of indigenous status	19,872 (84.6)	272,226 (87.0)	–2.4
Singleton birth	22,867 (97.4)	305,028 (97.5)	–0.1
Hospital birth	22,382 (95.3)	298,919 (95.5)	–0.2
Weight at birth, g			
<2,000	544 (2.3)	4,862 (1.6)	0.7
2,001–3,000	5,114 (21.8)	58,256 (18.6)	3.2†
3,001–4,000	14,550 (62.0)	205,289 (65.6)	–3.6†
4,001–5,000	2,005 (8.5)	31,705 (10.1)	–1.6
>5,001	43 (0.2)	469 (0.1)	0.1
Socioeconomic status			
Extremely advantaged	4,315 (18.4)	64,322 (20.6)	–2.2
Advantaged	3,365 (14.3)	43,709 (14.0)	0.3
Average	3,070 (13.1)	36,159 (11.6)	1.5
Disadvantaged	5,653 (24.1)	61,379 (19.6)	4.5†
Extremely disadvantaged	6,322 (26.9)	58,610 (18.7)	8.2†
Accessibility to services			
Highly accessible	14,926 (63.6)	199,090 (63.6)	0
Accessible	1,748 (7.4)	19,056 (6.1)	1.3
Moderately accessible	2,042 (8.7)	19,836 (6.3)	2.4
Remote	804 (3.4)	6,770 (2.2)	1.2
Very remote	2,812 (12.0)	13,808 (4.4)	7.6†
Ever hospitalized for a comorbidity‡	22,561 (96.1)	254,745 (81.4)	14.7†
Prior hospitalization‡	22,786 (97.0)	264,702 (84.6)	12.4†
Hospitalization in first year of life‡	18,361 (78.2)	189,959 (60.7)	17.5†
Hospitalization in first month of life‡	15,785 (67.2)	177,679 (56.8)	10.4†

*Percentage with history – percentage without history. †p<0.0001. ‡Excludes hospitalization for sequelae.

### Distribution of Sequelae

The highest rates of sequelae were observed for extragastrointestinal conditions: 2,407 and 977 per 100,000 person-years for those with and without prior enteric infection, respectively (Table 2). Intragastrointestinal sequelae, in comparison, occurred less frequently (400 and 226 per 100,000 person-years for those with and without prior enteric infection, respectively).

**Table 2:** Number and rates of first-time intragastrointestinal and extragastrointestinal sequelae for those with and without history of enteric infection, Western Australia, January 1, 1985–December 31, 2005*

Category of first-time sequelae	History		No history	Difference‡

No. (%)	Rate†	No. (%)	Rate†
Extragastroiintestinal sequelae	5,045 (100.0)	2,407.1		34,425 (100.0)	976.6	1,430.5
Respiratory infections	2,535 (50.2)	1,209.5		14,750 (42.8)	418.5	791.1
Infections of the middle ear and mastoid	1,544 (30.6)	736.7		13,164 (38.2)	373.5	363.2
Cellulitis, osteomyelitis, and myositis	364 (7.2)	173.7		2,327 (6.8)	66.0	107.7
Fever, unknown origin	303 (6.0)	144.6		1,409 (4.1)	40.0	104.6
Upper and lower urinary tract infections	78 (1.5)	37.2		715 (2.1)	20.3	16.9
Infections of lymphatic vessels	69 (1.4)	32.9		607 1.8)	17.2	15.7
Inflammatory diseases of the CNS	35 (0.7)	16.7		194 (0.6)	5.5	11.2
Arthropathies	39 (0.8)	18.6		275 (0.8)	7.8	10.8
Endometriosis	18 (0.4)	8.6		146 (0.4)	4.1	4.4
Orchitis and epididymitis	13 (0.3)	6.2		122 (0.4)	3.5	2.7
Bacteremia	8 (0.2)	3.8		55 (0.2)	1.6	2.3
Pilonidal cyst	38 (0.8)	18.1		600 (1.7)	17.0	1.1
Thyroiditis	1 (0.0)	0.5		2 (0.0)	0.1	0.4
Guillain-Barré syndrome	0	0.0		37 (0.1)	1.0	−1.0
Cardiac infections	0	0.0		22 (0.1)	0.6	−0.6
Intragastrointestinal sequelae	1,267 (100.0)	400.3		9,385 (100.0)	226.2	174.2
Intestinal obstruction, diverticular disease, irritable bowel syndrome/megacolon	278 (21.9)	87.8		1,536 (16.4)	37.0	50.8
Appendicitis	565 (44.6)	178.5		5,690 (60.6)	137.1	41.4
Infections of the oral cavity and esophagus	103 (8.1)	32.5		333 (3.5)	8.0	24.5
Enteritis, colitis and noninfective gastroenteritis	125 (9.9)	39.5		697 (7.4)	16.8	22.7
Infections of the stomach and duodenum	122 (9.6)	38.5		684 (7.3)	16.5	22.1
Peritonitis and ascites	16 (1.3)	5.1		25 (0.3)	0.6	4.5
Cholecystitis	40 (3.2)	12.6		346 (3.7)	8.3	4.3
Pancreatitis	18 (1.4)	5.7		74 (0.8)	1.8	3.9

*CNS, central nervous system. †Per 100,000 person-years. ‡Rate for patients with history – rate for patients without history.

### Risk for Hospitalization for First-time Sequelae According to Exposure Status

The rate of first-time hospitalization increased significantly for all outcomes analyzed; a slightly larger increase was found in risk for extragastrointestinal sequelae compared with intragastrointestinal sequelae (Table 3). Some of the elevation in the crude rate ratios was reduced after adjustment for sociodemographic and preexisting health status, which indicates confounding by these variables (Table A2), which indicates significant confounding for most of the variables assessed with respect to any and extragastrointestinal sequelae. However, for intragastrointestinal sequelae, only age, accessibility to services, born in hospital, mother’s region of birth, and hospitalization for a prior comorbidity significantly affected the risk for hospitalization for sequelae (Table A2). The adjusted rate ratios showed an increased rate of hospitalization for any sequela of 64%, intragastrointestinal sequelae of 52%, and extragastrointestinal sequelae of 63% for persons with prior enteric infection. The attributable risk fractions indicated that 39% of first-time hospitalizations for all sequelae (34% of intragastrointestinal and 39% of extragastrointestinal sequelae) were directly attributable to prior enteric infections.

**Table 3:** Number and rate of first-time hospitalizations, rate ratios, and attributable risk for sequelae for those with and without history of enteric infection, Western Australia, Australia, January 1,1985–December 31, 2000*

Type of sequelae	First-time hospitalizations					Crude rate ratio, RR (95% CI)	Adjusted† rate ratio, RR (95% CI)	Adjusted AR, %‡	Goodness of fit§
	With history			No history					
	No.	Rate		No.	Rate				
Any	5,634	27.8		41,054	11.8	2.36 (2.28–2.41)	1.64 (1.59–1.67)	39	0.05
Intragastrointestinal	1,267	4.0		9,385	2.3	1.77 (1.67–1.88)	1.52 (1.42–1.62)	34	0.04
Extragastrointestinal	5,045	24.1		34,425	9.8	2.46 (2.39–2.54)	1.63 (1.57–1.68)	39	0.08

*Rates are per 100,000 person-years. RR, relative risk; CI, confidence interval; AR, attributable risk. †Multivariate Cox regression estimating the adjusted rate ratio of first-time hospitalization for any, intragastrointestinal, and extragastrointestinal sequelae. Adjusted for gender, indigenous status, year of birth, age at exposure or proxy, singleton, weight at birth, hospital birth, mother’s region of birth, father’s region of birth, socioeconomic status, accessibility to services and previous hospitalization for comorbidity. ‡Proportion of first-time hospitalizations for sequelae where previous exposure to an enteric infection was a component cause. §Pseudo R^2^. As explained by Hosmer and Lemeshow (*20*), a measure analogous to R^2^ would be useful as a measure of Cox regression model performance; however, although a pseudo R^2^ can be calculated the values obtained are often low because of the censored nature of the data even though the model is adequate. In our models the R^2^ values were 0.05, 0.04 and 0.08 for the 3 models (any, intragastrointestinal, and extragastrointestinal), respectively. The models generated were population-based descriptive models, which aimed to evaluate the average effect on survival to first-time hospitalization with the outcome of interest adjusted for known and measurable confounders, rather than predict the probability of survival for a specified individual. Thus, the most important assessment criteria for evaluating the appropriateness of a descriptive Cox regression model is that the proportional hazards assumption is not violated and the overall model is significant. In all of our models the proportional hazards assumption was tested and found not to be violated and the overall model significance was Prob > χ^2^ <0.00005.

### Survivor Profile

The survivor profiles for extragastrointestinal and intragastrointestinal sequelae differed (Figure). Extragastrointestinal sequelae occurred predominantly in the first 5 years after a first-time enteric infection; thereafter, the survivor function curves did not change significantly for the 2 groups. In contrast, the survivor function curves for intragastrointestinal sequelae indicate little difference between those with and without prior enteric infection over the first 10 years. After this time the survivor function curves deviate significantly, suggesting that these sequelae mostly occur later.

**Figure:**
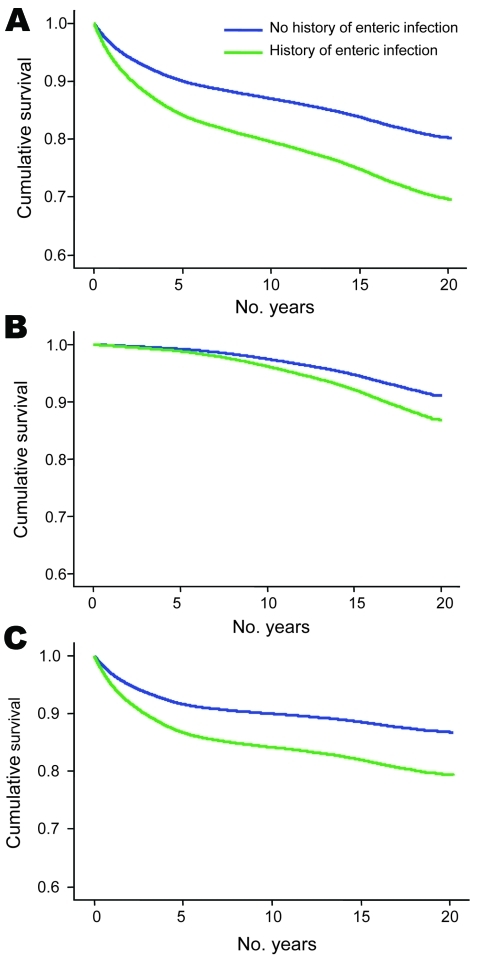
Kaplan-Meier estimates of the adjusted survivor function with respect to any sequelae (A), intragastrointestinal sequelae (B), and extragastrointestinal sequelae (C) for those with and without prior enteric infection, Western Australia, Australia, January 1, 1985–December 31, 2000.

### Type of Enteric Notifications and Risk for Sequelae by Type of Infection

Of the 23,477 first-time notifications of enteric infection, the most frequently reported single defined causes were *Campylobacter* spp. (17%), viruses (17%), and *Salmonella* spp. (12%). Forty-two percent of notifications were identified solely by using hospitalization records as “Enteritis and gastroenteritis, not otherwise specified.” An additional 12% of notifications were of various other specific organisms. The risk profile for intragastrointestinal and extragastrointestinal sequelae were similar for the most common infective pathogens (Table 4). The exception was for *Salmonella* infection, which did not confer an increased risk for intragastrointestinal sequelae.

**Table 4:** Crude and adjusted rate ratio and attributable risk for first-time hospitalization for sequelae, by type of prior infection and classification of sequelae, Western Australia, Australia, January 1,1985–December 31, 2000*

Type of sequelae and type of prior infection	Crude rate ratio, RR (95% CI)	Adjusted† rate ratio, RR (95% CI)	Adjusted AR, %‡	Goodness of fit§
Any				0.05
Campylobacteriosis	1.56 (1.43–1.69)	1.52 (1.39–1.66)	34	
Giardiasis	2.10 (1.91–2.31)	1.51 (1.36–1.68)	34	
Salmonellosis	1.86 (1.71–2.03)	1.39 (1.26–1.53)	28	
Viral enteritis	2.10 (1.96–2.24)	1.68 (1.56–1.81)	40	
Enteritis/gastroenteritis NOS	2.60 (2.51–2.70)	1.76 (1.70–1.84)	43	
Extragastrointestinal				0.07
Campylobacteriosis	1.50 (1.37–1.64)	1.45 (1.32–1.60)	31	
Giardiasis	2.26 (2.04–2.49)	1.54 (1.38–1.73)	35	
Salmonellosis	2.01 (1.85–2.21)	1.43 (1.29–1.58)	31	
Viral enteritis	2.12 (1.98–2.27)	1.63 (1.51–1.76)	39	
Enteritis/gastroenteritis NOS	2.69 (2.58–2.80)	1.74 (1.66–1.82)	43	
Intragastrointestinal				0.02
Campylobacteriosis	1.76 (1.51–2.05)	1.64 (1.40–1.93)	39	
Giardiasis	1.37 (1.10–1.68)	1.29 (1.03–1.61)	23	
Salmonellosis	1.09 (0.87–1.34)	1.00 (0.79–1.25)	0	
Viral enteritis	1.99 (1.72–2.29)	1.56 (1.34–1.85)	36	
Enteritis/gastroenteritis NOS	2.03 (1.88–2.20)	1.66 (1.52–1.81)	40	

*RR, relative risk; CI, confidence interval; AR, attributable risk; NOS, not otherwise specified. †Multivariate Cox regression estimating the adjusted rate ratio of first-time hospitalization for any, intragastrointestinal, and extragastrointestinal sequelae. Adjusted for gender,indigenous status, year of birth, age at exposure or proxy, singleton, weight at birth, hospital birth, mother’s region of birth, father’s region of birth, socioeconomic status, accessibility to services, and previous hospitalization for comorbid conditions. ‡Proportion of first-time hospitalizations for sequelae where previous exposure to the specified enteric infection was a component cause. § Pseudo R^2^. In our models, the R^2^ values were 0.05, 0.07, and 0.02 for the 3 sets of models (any, extragastrointestinal, and intragastrointestinal) respectively. In all models the proportional hazards assumption was tested and found not to be violated, and the overall model significance was Prob > χ2 <0.00005.

## Discussion

Our study showed that prior exposure to an enteric infection during childhood or adolescence increases the risk for a first-time hospitalization for a wide range of intragastrointestinal and extragastrointestinal illnesses by 64% over 22 years of follow-up. Furthermore, 39% of first-time hospitalizations for these illnesses were directly attributable to a previous enteric infection. The risk for extragastrointestinal sequelae was higher than that of intragastrointestinal sequelae, and the time of onset for the 2 categories of sequelae differed. Intragastrointestinal sequelae occurred much later after exposure than extragastrointestinal sequelae, a finding that has not been reported previously.

We found that respiratory and middle ear infections were the largest contributors to the excess rate of extragastrointestinal conditions. Appendicitis, the most common intragastrointestinal sequelae in this age-group, had a 23% increased risk in persons previously exposed to enteric infection. This association has previously been suggested for *Campylobacter* infection (*9*). We also found a 57% increase in risk for enteritis, colitis, and noninfective gastroenteritis, a diagnostic grouping that includes ulcerative colitis and Crohn disease; again, this association has previously been made (*7*,*8*). However, contrary to previous studies that reported an association between enteric infection and subsequent disease (*14*,*21*–*26*), the findings in our study suggest that risk increases over time, especially with regards to intragastrointestinal sequelae where the risk becomes greatest >10 years after onset of gastroenteritis infections in children and adolescents.

Our findings indicate that being male, indigenous, of low birthweight, and socioeconomically disadvantaged and being born outside the metropolitan area increases risk for developing the measured sequelae in exposed and unexposed persons (data not shown). However, we confirmed that previous exposure to an enteric infection is an important risk factor because after these factors are adjusted for, a 64% increase in risk for sequelae remains. We found that salmonellosis afforded no additional risk for intragastrointestinal sequelae but increased the risk for extragastrointestinal sequelae by 43%. With this exception, all common causes of infective gastroenteritis were equally represented as increasing the risk for sequelae.

This study has several strengths and limitations that warrant consideration when interpreting our results. A major strength of the research was the use of linked birth, hospital, death, and communicable disease notifications data obtained over a long time, which made available a comprehensive patient-based longitudinal dataset, as opposed to an events-based dataset. Data were of a routine administrative nature, and there was no likelihood of a Hawthorne effect (changes caused by participants being observed) or recall bias. The study covered the entire population of those born in Western Australia and therefore avoided challenges to external validity that arise when patient series are reported from selected institutions.

Our study incorporated an extensive amount of sociodemographic and other health-related data, thus enabling adjustment of the models for a range of measurable confounding variables. We recognize that a variety of unmeasurable, potentially confounding, sociodemographic factors may not have been completely captured by this study. However, given the magnitude of the increase in risk for sequelae observed for those exposed, all of this increase in risk would be unlikely to be removed if these potential confounders could be adjusted for. One way to have adjusted for unmeasurable factors would have been to restrict the study population to those hospitalized for a condition unrelated to the exposure (prior enteric infection) and the outcome, such as injury. However, this adjustment would have dramatically reduced the sample size, and hence the power of the study, and would have substantially limited generalizability.

Substantial errors in our dataset are unlikely because classification regimens were applied consistently throughout; validation research on the WADLS (*27*) has shown that missing data items are uncommon (<1%) and that the technical performance of the linkage between records is high (>99% specificity and sensitivity). Accordingly, our data are a highly robust representation of the population studied, based on usual hospital admission practices and outcomes of persons after notification of an enteric infection within the Australian healthcare system.

Our study relied on the use of notifications or hospitalization for enteric infections; not all enteric infections are notified. Enteric infections that cause only mild symptoms may go unreported either because the person may not seek medical advice or because a medical practitioner may not undertake confirmatory testing (*28*). Our conclusions are based on an assumption that a similar proportion of underreporting of enteric infection occurred for those classified with and without prior infection. Because notification of enteric infection is more likely for moderate to severe illness, our results pertain to this group rather than all enteric infections. This restriction limits generalizability (the range of persons not studied directly to whom the results can be applied), although it does not affect the validity of our results.

Sequelae in this study were captured as a first-time hospitalization for the condition of interest. Thus, some sequelae that produced only mild symptoms not requiring hospitalization were not captured, producing an underestimation of first-time sequelae. We evaluated the risk for first-time hospitalization for sequelae rather than incidence of sequelae per se*.* Although this type of evaluation is a limitation, the sequelae that have been measured are of clinical importance by virtue of the need for hospitalization.

We used the Western Australian birth register as the method of identification. These data enabled comprehensive inclusion of all persons born in the state, enabling complete capture of first-time enteric infections and outcome, within the definitions discussed above. The birth register also provided information about parents’ place of birth, enabling these potential confounding variables to be evaluated when constructing the models. However, the use of a birth cohort did not permit the inclusion of international or interstate migrants. Again, this exclusion potentially limits the generalizability of our findings because migrants may have different risks of developing sequelae than nonmigrants. Inclusion of migrants in this study would have created difficulty capturing exposure and outcome data because their health records were not available before individuals became residents of Western Australia.

Although our study estimated the magnitude of the increased risk for illness attributable to prior enteric infections, we cannot explain why enteric infection during childhood or adolescence subsequently increases the risk for illness. Genetic susceptibility for disease, combined with an environmental trigger, has previously formed the basis for explaining this risk for conditions with an autoimmune basis, such as inflammatory bowel disease (*15*,*21*). Our finding of an increased risk for nongastrointestinal infections is more difficult to account for by autoimmune mechanisms. We speculate that increased risk for nongastrointestinal infections may result from dysregulated immunity, particularly in the short to medium term, after gastroenteritis, brought about by the influence of enteric infection on the gastrointestinal immune system.

Medium- to long-term adverse health implications of gastroenteritis must be accurately assessed so that appropriate risk-management strategies can be developed for those exposed to enteric infections. Recent reports have described the effect of acute gastrointestinal infections in industrialized countries, such as Australia (*2*,*28*) and Canada (*29*). These investigations have focused on identifying rates of, and risk factors for, infectious gastrointestinal illness with the goal of informing public health policy and planning. The need to investigate the impact of long-term sequelae of infectious gastrointestinal illness has also been highlighted (*29*). According to our data, sequelae are clearly substantial and provide another reason for trying to reduce the incidence of acute gastrointestinal infection worldwide.

Our study shows that enteric infection during childhood or adolescence increases the risk for first-time hospitalization for a range of intragastrointestinal and extragastrointestinal disease for 2 decades after onset of infection. This risk is greater, and occurs earlier, for extragastrointestinal sequelae than for intragastrointestinal sequelae. Our results highlight the importance of identifying ways of reducing such infections.

## Appendix

**Table A1:** Classification of intragastrointestinal and extragastrointestinal sequelae in children and young adults after infective gastroenteritis*

Condition	ICD-9-CM	ICD-10-AM
Intragastrointestinal conditions		
Cellulitis and abscess of mouth, oesophagitis	528.3, 530.1	K12.2, K20
Gastritis (excluding alcoholic gastritis), duodenitis, enteritis, colitis (excluding irradiation colitis), appendicitis	535 (excluding 535.30), 540, 541, 542, 555, 556	K29 (excluding K29.2), K35, K50, K51, K52
Intestinal obstruction, irritable colon, megacolon, peritonitis, diverticulitis	56-0, 562, 564.1, 564.7 567	K56, K57, K58, K59, K65
Cholelithiasis, acute cholecystitis, cholangitis, pancreatitis, ascites	574, 575.1, 576.1, 577.0, 577.1, 789.5	K80 (excluding K80.2& K80.5), K81, K83.0, K85, K86.1,R18
Extragastrointestinal conditions		
Thyroiditis	245	E06
Meningitis, encephalitis, myelitis, encephalomyelitis, intracranial and intraspinal abscesses, phlebitis and thrombophlebitis of intracranial venous sinuses.	320, 321, 322, 323, 324, 325.	G00-G08.
Guillain-Barré syndrome	357.0	G61.0
Otitis media, mastoiditis	381, 382, 383	H65-H67, H70,
Pericarditis, endocarditis, myocarditis	420, 421,422	I30, I32, I38, I39, I40, I41
Mesenteric lymphadenitis, pilonidal cyst	685	I88.0, L05
Sinusitis, pharyngitis, tonsillitis, laryngitis, tracheitis, acute upper respiratory infection, acute bronchitis, bronchiolitis, pneumonia, influenza	461-466, 480-487	J01-J06, J20-J22, J10-J18
Glomerulonephritis, infections of kidney, cystitis (excluding irradiation), orethritis (excluding sexually transmitted), orchitis and epididymitis	580, 582, 590, 595 (excluding 595.82), 597, 604	N00, N001, N003, N005, N10-N13.5, N30 (excluding N30.4), N34, N45
Endometriosis	617	N80
Cellulitis, osteomyelitis, fever of unknown origin, myositis	681–682, 730, 780.6	L03, M60, M86, R50
Arthropathy associated with infections, hyogenic arthritis, arthritis in other bacterial diseases, postdysenteric arthropathy, Reiter syndrome, reactive arthropathies, postinfection arthropathies	99.3, 711, 716	M00.0–M00.9, M01.3, M02.1, M02.3, M02.8, M02.9, M03.2, M03.6
Llymphoma and other named immunoproliferative small intestinal diseases	200.8, 203.8	C88.3
Bacteremia	790.7	A49.9

*ICD-9-CM, International Classification of Diseases, 9th Revision, Clinical Modification; ICD-10-AM, International Classification of Diseases, 10th Revision, Australian Modification. Any sequelae classification includes all conditions listed in the table.

**Table A2:** Significance, hazard ratios, and 95% CIs of variables in the 3 models measuring sequelae in children and young adults after infective gastroenteritis*

Variables in model	Category	Any sequelae		Extragastrointestinal sequelae

p value	HR (95% CI)	p value	HR (95% CI)
Previous history of an enteric infection	Yes	<0.0001	1.642 (1.590–1.695)		<0.0001	1.628 (1.574–1.684)
Sex	Female	<0.0001	0.806 (0.790–0.822)		<0.0001	0.789 (0.772–0.806)
Indigenous status	Aboriginal	<0.0001	1.807 (1.728–1.890)		<0.0001	1.897 (1.810–1.988)
Singleton birth	Yes	0.0071	1.090 (1.024–1.160)		0.0084	1.094 (1.023–1.170)
Year of birth		<0.0001	0.978 (0.976–0.981)		<0.0001	0.989 (0.986–0.991)
Age in years at enteric infection or proxy		<0.0001	0.872 (0.868–0.877)		<0.0001	0.810 (0.805–0.815)
Weight at birth, g	<2,000	<0.0001	1.000		<0.0001	1.000
	2,001–3,000	<0.0001	0.799 (0.747–0.854		<0.0001	0.768 (0.715–0.824)
	3,001–4,000	<0.0001	0.745 (0.698–0.796)		<0.0001	0.706 (0.658–0.756)
	4,001–5,000	<0.0001	0.728 (0.678–0.782)		<0.0001	0.686 (0.636–0.740)
	>5,001	0.0747	0.809 (0.641–1.021)		0.0871	0.808 (0.633–1.032)
Socioeconomic status (SEIFA)	Extremely advantaged	<0.0001	1.000		<0.0001	1.000
	Advantaged	0.1977	1.022 (0.989–1.057)		0.1657	1.026 (0.989–1.064)
	Average	0.0003	1.066 (1.029–1.103)		0.0005	1.069 (1.029–1.111)
	Disadvantaged	<0.0001	1.143 (1.110–1.177)		<0.0001	1.166 (1.129–1.204)
	Extremely disadvantaged	<0.0001	1.112 (1.079–1.146)		<0.0001	1.122 (1.086–1.159)
Accessibility to services	Highly accessible	<0.0001	1.000		<0.0001	1.000
	Accessible	<0.0001	1.183 (1.141–1.227)		<0.0001	1.203 (1.157–1.252)
	Moderately accessible	<0.0001	1.218 (1.175–1.262)		<0.0001	1.257 (1.210–1.306)
	Remote	<0.0001	1.329 (1.260–1.402)		<0.0001	1.410 (1.333–1.492)
	Very remote	<0.0001	1.149 (1.102–1.197)		<0.0001	1.206 (1.154–1.260)
Born in hospital	Yes	<0.0001	0.883 (0.838–0.931)		<0.0001	0.820 (0.776–0.867)
Mother’s region of birth	Australia	<0.0001	1.000		<0.0001	1.000
	New Zealand	0.0006	0.852 (0.778–0.934)		0.0108	0.880 (0.798–0.971)
	United Kingdom	0.0018	0.925 (0.881–0.972)		0.0131	0.935 (0.886–0.986)
	North America	0.1076	0.833 (0.668–1.041)		0.1996	0.853 (0.668–1.088)
	Europe	0.0101	0.886 (0.808–0.972)		0.0106	0.874 (0.788–0.969)
	Eastern Europe, former USSR, and Baltic States	0.3687	0.897 (0.709–1.136)		0.5647	0.926 (0.711–1.204)
	Africa	0.1407	0.912 (0.808–1.031)		0.2897	0.931 (0.816–1.063)
	Central/South America and Caribbean	0.7729	0.964 (0.754–1.233)		0.4821	0.906 (0.687–1.194)
	Melanesia, Micronesia, and Polynesia	0.2428	0.804 (0.558–1.159)		0.2646	0.796 (0.534–1.188)
	Asia	<0.0001	0.593 (0.545–0.645)		0.0000	0.600 (0.546–0.659)
	Middle East	0.3621	0.892 (0.699–1.140)		0.2494	0.855 (0.655–1.116)
Father’s region of birth	Australia	<0.0001	1.000		<0.0001	1.000
	New Zealand	0.0989	0.950 (0.893–1.010)		0.2285	0.960 (0.899–1.026)
	United Kingdom	0.0580	1.033 (0.999–1.069)		0.1773	1.026 (0.989–1.065)
	North America	0.1267	0.910 (0.806–1.027)		0.1727	0.912 (0.799–1.041)
	Europe (N,S, and W)	0.0239	0.934 (0.881–0.991)		0.0083	0.915 (0.857–0.977)
	Eastern Europe, former USSR, and Baltic States	0.1320	0.853 (0.693–1.049)		0.0285	0.768 (0.606–0.973)
	Africa	0.0894	0.924 (0.843–1.012)		0.1606	0.931 (0.843–1.029)
	Central/South America and Caribbean	0.8662	1.019 (0.822–1.261)		0.8923	1.016 (0.803–1.286)
	Melanesia, Micronesia, and Polynesia	0.3944	0.906 (0.721–1.138)		0.5997	0.937 (0.733–1.196)
	Asia	<0.0001	0.847 (0.785–0.914)		<0.0001	0.825 (0.758–0.897)
	Middle East	0.1435	0.857 (0.697–1.054)		0.3362	0.897 (0.718–1.120)
Hospitalization for a concurrent illness	Yes	<0.0001	0.814 (0.766–0.864)		<0.0001	6.671 (5.637–7.893)

*HR, hazard ratio; CI, confidence interval; USSR, Union of Soviet Socialist Republics; –, not applicable.

**Appendix:** Table 3. Model for measuring intragastrointestinal sequelae in children and young adults after infective gastrointeritis*

Variables in model	Category	p value	HR (95% CI)
Previous history of an enteric infection	Yes	<0.0001	1.517 (1.421–1.621)
Age, y, at enteric infection or proxy	–	<0.0001	1.121 (1.113–1.129)
Accessibility to services	Highly accessible	<0.0001	1.000
	Accessible	0.0024	1.112 (1.038–1.190)
	Moderately accessible	0.0394	1.073 (1.003–1.148)
	Remote	0.5996	0.971 (0.868–1.085)
	Very remote	0.0002	0.858 (0.792–0.930)
Born in hospital	Yes	<0.0001	0.750 (0.663–0.849)
Mother’s region of birth	Australia	<0.0001	1.000
	New Zealand	0.0001	0.708 (0.594–0.845)
	United Kingdom	0.0121	0.901 (0.831–0.978)
	North America	0.2124	0.751 (0.479–1.178)
	Europe (N,S, and W)	0.1709	0.896 (0.766–1.048)
	Eastern Europe, former USSR, and Baltic States	0.3864	0.860 (0.611–1.210)
	Africa	0.0233	0.761 (0.602–0.964)
	Central /Southern America and Caribbean	0.1062	1.338 (0.940–1.903)
	Melanesia, Micronesia, and Polynesia	0.4165	0.735 (0.351–1.543)
	Asia	<0.0001	0.575 (0.503–0.657)
	Middle East	0.3952	0.860 (0.608–1.217)
Ever had a hospitalization for a concurrent illness	Yes	<0.0001	1.847 (1.580–2.158)

*HR, hazard ratio; CI, confidence interval; –, not applicable. Model for intragastrointestinal sequelae was significantly different from other models.
